# Supplementary material for: Expert decision-making in clinicians: An auto-analytic ethnographic study of operational decision-making in urgent care
Source: PLoS One. 2025 Jan 22;20(1):e0311748. doi: 10.1371/journal.pone.0311748 (PMC11753633; doi:10.1371/journal.pone.0311748)
Supplement: Attachment — Submitted filename: Response to Reviewers.docx [file pone.0311748.s001.docx]

Yunus Centre for Social Business and Health

Glasgow Caledonian University

Glasgow G4 0BA

27^th^ August 2024

Dear PloS One editorial Team, Reviewers 1 and 2,

**Re: PONE-D-24-02400**

**Expert decision-making in clinicians: An auto-analytic ethnographic study of operational decision-making in urgent care. PLOS ONE**

We thank the reviewers for their very helpful and constructive feedback on our article. We also thank them for supportive comments about the importance of research in this field and the methodology employed.

We have reviewed the comments and agree with all points made. We have amended our manuscript to reflect the recommendations.

Please see below our response to each of the comments made, how and where these have been addressed in the ‘track changes’ version of our manuscript. We have also included this as a table in case this is more helpful for the reviewers to use in second review.

Yours faithfully

Dr Nicola Irvine

**Reviewer #1:** 
Abstract:
1. It is important to briefly describe the setting of study since it influences readers understanding of the study.

*We have updated the original manuscript to include a brief description of the urgent care facility, setting, and population (lines 29-30).*

2. As your context is specific, I think your study is focused Ethnography.

*We have updated the description of methods in the abstract to incorporate the reviewers recommendations of the term ‘Focused Ethnography’ (line 27-28).* *As a crucial component of the study, the analytic autoethnographic component is also included.*

Methods:
1. Triangulation in method of data gathering, data sources and investigators is necessary. Please declare them as your limitations if they are not committed in your study.

*A limitations section has been added to the manuscript. This explains the limits of the triangulation that was possible. Lines 590-592*

2. Setting of the ward including number of beds, physicians, private or public, exact duration of spending time in the ward for study is necessary.

3. Diverse background of participants should be pointed out by more details.

4. You should have thick description in fact you should explain about relationships and events in addition to physical environment.

*We have provided a richer description of the ward setting as advised. This includes the resources, types of patients, relationships, and events pertinent to the study Lines 153 – 196*

*The duration of time spent has been added to the data collection section at Lines 214-217*

*Participants descriptions have been updated to provide more details Lines 198-206*

5. Detailed explanations about interviews are needed including time, being face-to-face and etc.

*A fuller description of the particulars of the interviews and notes taken has been added Lines 217-238*

6. Was data analysis according to inductive or deductive methods?

*Description of the Gioia Methodology of analysis has been updated to specify inductive analysis Lines 251-266.*

7. Describe about considerations of rigor and also triangulation in analysis.

*Methodology section has been updated to improve descriptions of rigor in the method and sources for triangulation. Limitations in triangulation added to end of discussion as described above Lines 120-139*

8. Please describe that data collection and data analysis happened simultaneously.

*Manuscript adjusted to explain simultaneous collection and analysis of data Line 147*

9. Conducting focus group should be described by more details including number of FGs, participants, time, questions, etc.

10. Please describe about participants characteristics.

*Description of the focus group has been updated to include more detail as recommended including the participant characteristics Lines 268-285*

Discussion:
Please explain about limitations of your work like possibility of social desirability bias or other biases.

*Line 588-614 Limitations section added and include biases as recommended by the reviewer*

**Reviewer #2:** 
The manuscript can be further enhanced if the authors consider the following comments:

1- Clarify the methodological details, particularly how the Gioia method was applied, to help readers better understand the thematic analysis and model development process.

*Lines 248-266 Methodology section updated as recommended*

2- Provide a broader contextualization by comparing the findings with decision-making processes in other high-stakes domains, strengthening the argument that intuitive decision-making is a universal skill among experts.

*Lines 572-586 Expansion on discussion of intuitive expertise in other domains*

3-Discuss the study's limitations in more detail, including the small sample size and potential bias from the lead author’s dual role. Address the generalizability of the findings to other settings and specialties.

*Line 588-614 Limitations section added and include biases as recommended by the reviewer*

4- Expand on future research directions, particularly in developing training programs or AI systems, providing specific suggestions for experimental studies or simulations to build on these findings.

*We thank the reviewer for this suggestion. Indeed, we did initially consider including more detail about how our work could inform future research into AI systems for decision support in this domain. However, on further reading we realised it is an expansive topic in which we are to advise upon re: methodology of future studies. We acknowledge our limitations in being able to expand upon this further in the final section (Line 629-633)*

| Section | Reviewer 1 | Reviewer 2 | Authors’ response | Position in marked up manuscript |
| --- | --- | --- | --- | --- |
| Abstract | 1. It is important to briefly describe the setting of study since it influences readers understanding of the study |  | We updated the original manuscript to include a brief description of the urgent care facility, setting, and population | Lines 29-30 |
|  | 2. As your context is specific, I think your study is focused Ethnography. |  | We have updated the description of methods in the abstract to incorporate the reviewers recommendations of the term ‘Focused Ethnography’ | Line 27-28 |
| Methods | 1. Triangulation in method of data gathering, data sources and investigators is necessary. Please declare them as your limitations if they are not committed in your study |  | A limitations section has been added to the manuscript. This explains the limits of the triangulation that was possible | Lines 590-592 |
|  | 2. Setting of the ward including number of beds, physicians, private or public, exact duration of spending time in the ward for study is necessary. |  | We have provided a richer description of the ward setting as advised. This includes the resources, types of patients, relationships, and events pertinent to the study | Lines 153 – 196 |
|  | 4. You should have thick description in fact you should explain about relationships and events in addition to physical environment |  | The duration of time spent has been added to the data collection section | Lines 214-217 |
|  | 3. Diverse background of participants should be pointed out by more details |  | Participants descriptions have been updated to provide more detail | Lines 198-206 |
|  | 5. Detailed explanations about interviews are needed including time, being face-to-face and etc |  | A fuller description of the particulars of the interviews and notes taken has been added | Lines 217-238 |
|  | 6. Was data analysis according to inductive or deductive methods? | 1- Clarify the methodological details, particularly how the Gioia method was applied, to help readers better understand the thematic analysis and model development process. | Description of the Gioia Methodology of analysis has been updated to specify inductive analysis | Lines 248-266 |
|  | 7. Describe about considerations of rigor and also triangulation in analysis |  | Methodology section has been updated to improve descriptions of rigor in the method and sources for triangulation. | Lines 120-139 |
|  |  |  | Limitations in triangulation added to end of discussion as described above | Lines 590-592 |
|  | 8. Please describe that data collection and data analysis happened simultaneously |  | Manuscript adjusted to explain simultaneous collection and analysis of data | Line 147 |
|  | 9. Conducting focus group should be described by more details including number of FGs, participants, time, questions, etc. |  | Description of the focus group has been updated to include more detail as recommended including the participant characteristics | Lines 268-285 |
|  | 10. Please describe about participants characteristics |  |  |  |
| Discussion | Please explain about limitations of your work like possibility of social desirability bias or other biases | 3-Discuss the study's limitations in more detail, including the small sample size and potential bias from the lead author’s dual role. Address the generalizability of the findings to other settings and specialties | Limitations section added and include biases as recommended by the reviewers, the issue of sample size, and generalisability | Line 588-614 |
|  |  | 2- Provide a broader contextualization by comparing the findings with decision-making processes in other high-stakes domains, strengthening the argument that intuitive decision-making is a universal skill among experts | We have expanded upon our discussion of intuitive expertise in other domains | Lines 572-586 |
|  |  | 4- Expand on future research directions, particularly in developing training programs or AI systems, providing specific suggestions for experimental studies or simulations to build on these findings | Thank you for this suggestion. We did consider including more detail about how our work could inform AI systems, however, this is a very large topic to address. We acknowledge our limitations in being able to expand upon this in the final section. Given our limited knowledge we are unqualified to make more specific recommendations on methodology to explore this in detail in future | Lines 629-633 |
